# Supplementary material for: Performance evaluation of developed macrophyte-assisted vermifiltration system designed with varied macrophytes and earthworm species for domestic wastewater treatment
Source: PLoS One. 2023 Mar 1;18(3):e0281953. doi: 10.1371/journal.pone.0281953 (PMC9977024; doi:10.1371/journal.pone.0281953)
Supplement: Attachment — Submitted filename: Response to Reviewers-...docx [file pone.0281953.s002.docx]

Rebuttal letter

Journal Requirements:

When submitting your revision, we need you to address these additional requirements.

1. Please ensure that your manuscript meets PLOS ONE's style requirements, including those for file naming. The PLOS ONE style templates can be found at https://journals.plos.org/plosone/s/file?id=wjVg/PLOSOne_formatting_sample_main_body.pdf and 
https://journals.plos.org/plosone/s/file?id=ba62/PLOSOne_formatting_sample_title_authors_affiliations.pdf

**Authors’ Response: Authors have addressed these additional requirements by crosschecking to make our revision meet PLOS ONE's style requirements, including those for file naming.**

2. Thank you for stating the following in the Acknowledgments Section of your manuscript:

"The project was funded by the Government of Ghana Research fund with the credit number 4646-GH. Authors would like to acknowledge all technicians at the Environmental Quality Engineering (EQE) Laboratory of the Department of Civil Engineering, KNUST who provided laboratory and technical assistance throughout the study"

We note that you have provided funding information that is not currently declared in your Funding Statement. However, funding information should not appear in the Acknowledgments section or other areas of your manuscript. We will only publish funding information present in the Funding Statement section of the online submission form. 
Please remove any funding-related text from the manuscript and let us know how you would like to update your Funding Statement. Currently, your Funding Statement reads as follows:

"The project was funded by the Government of Ghana Research fund with the credit number 4646-GH"

Please include your amended statements within your cover letter; we will change the online submission form on your behalf.

**Authors’ Response: Authors have responded to the error identified by journal on the Funding Statement in the Acknowledgments Section. Authors have removed any funding-related text from the manuscript and have corrected this error in the revised manuscript submission.**

3. "In your Data Availability statement, you have not specified where the minimal data set underlying the results described in your manuscript can be found. PLOS defines a study's minimal data set as the underlying data used to reach the conclusions drawn in the manuscript and any additional data required to replicate the reported study findings in their entirety. All PLOS journals require that the minimal data set be made fully available. For more information about our data policy, please see http://journals.plos.org/plosone/s/data-availability.

"Upon re-submitting your revised manuscript, please upload your study’s minimal underlying data set as either Supporting Information files or to a stable, public repository and include the relevant URLs, DOIs, or accession numbers within your revised cover letter. For a list of acceptable repositories, please see http://journals.plos.org/plosone/s/data-availability#loc-recommended-repositories. Any potentially identifying patient information must be fully anonymized.

Important: If there are ethical or legal restrictions to sharing your data publicly, please explain these restrictions in detail. Please see our guidelines for more information on what we consider unacceptable restrictions to publicly sharing data: http://journals.plos.org/plosone/s/data-availability#loc-unacceptable-data-access-restrictions. Note that it is not acceptable for the authors to be the sole named individuals responsible for ensuring data access.

We will update your Data Availability statement to reflect the information you provide in your cover letter."

Please review your reference list to ensure that it is complete and correct. If you have cited papers that have been retracted, please include the rationale for doing so in the manuscript text, or remove these references and replace them with relevant current references. Any changes to the reference list should be mentioned in the rebuttal letter that accompanies your revised manuscript. If you need to cite a retracted article, indicate the article’s retracted status in the References list and also include a citation and full reference for the retraction notice.

**Authors’ Response: Authors have provided and uploaded the minimal data set underlying the results as supporting information files during the re-submission of our revised manuscript.**

Reviewers' comments:

Reviewer #1: Authors describe the use of various macrophytes and earthworm species in the effort for doing vermifiltration in WWTP. Several suggestions can be addressed below:
1. Abstract seems to be too qualitative. Authors are suggested to provide more quantitative insight.

**Authors’ Response: Authors have provided Quantitative insights in the Abstract of the revised manuscript in Line 38, Line 39, Line 41 and Line 45.**

2. Line 86 and below in introduction are suggested to be separated as new paragraph.
**Authors’ Response: Authors agree; Line 86 and below in introduction have been separated as new paragraph in the revised manuscript.**

3. Experimental set up has no reference

**Authors’ Response: References have been provided in the revised manuscript of the Experimental set up in Line 126, Line 128, Line 131 and Line 137. The references provided are;**

**1. Francesconi, K. A., Gailer, J., Edmonds, J. S., Goessler, W., & Irgolic, K. J. Uptake of arsenic-betaines by the mussel Mytilus edulis. Comparative Biochemistry and Physiology, 1999, 122, 131–137.**

**2. Yu, J., Xiong, M., Ye, S., Li, W., Xiong, F., Liu, J. and Zhang, T., Effects of stocking density and artificial macrophyte shelter on survival, growth and molting of jevenile red swamp crayfish (Procambarus clarkii) under experimental conditions. Aquaculture, 2020, 521, 735001.**

4. Authors need to conduct various test such as homogenity, normality, and independency before performing anova.

**Authors’ Response: Authors conducted chi-square tests of homogeneity and Shapiro-Wilk normality to determine the homogeneous, parametric and interdependency nature of the data, before performing ANOVA. This has been stated in the Statistical analysis section of the revised manuscript in Line 199 to Line 201.**

5. There are many inconsistencies in writings and also syntax errors. Authors are suggested to proofread the manuscript by the help of native English professional.

**Authors’ Response: Authors have proofread the manuscript with the help of native English professional and have corrected all inconsistencies in writings and syntax errors.**

Reviewer #2: (1) On the Experimental setup
(a) It is not clear why a hydraulic loading rate (HLR) is provided or its significance

**Authors’ Response: Authors have provided the significance of providing a hydraulic loading rate (HLR) in Line 144 to 146, and Line 444 to 445.**

(b) It is not clear why the Effluent samples were collected every 48 hours and for two weeks

**Authors’ Response: Effluent samples were collected every 48 hours and for two weeks to provide the maximum stabilization conditions for effective analysis. This sampling procedure was used and established in Nsiah-Gyambibi et al. (2022) and Awuah (2006). These established references have been provided in Line 148 to 149 in the revised manuscript.**

(c) It is not clear what the stage of growth / growth characteristics of the macrophytes were after establishment in the experimental treatment units
**Authors’ Response: Authors have provided the stocking area of the macrophytes in Line 129 to 130. Authors however deemed the growth characteristics of the macrophytes had little contribution to the outcomes and purpose of this laboratory scaled study due to the sampling method chosen. Nevertheless, in our future and subsequent development of this study where direct field experiments will be combined with the laboratory scaled units in an extended sampling method, the growth characteristics of the macrophytes would be adequately investigated.**

(2) On the results section on the expression of pathogen and helminth characteristics of wastewater and Pathogen count in effluents:
(a) The units are indicated as cfu100 mgL-1 . Why not the conventional CFU 100 ml-1.?

**Authors’ Response: Authors agree with reviewer. Authors have corrected all cfu100 mgL^-1^ into the conventional CFU 100 ml^-1^ in the revised manuscript.**

(b) Consider using the log unit for pathogen count – this is convenient when working with wastewater

**Authors’ Response: Authors have considered using the log unit for pathogen count. All pathogen count have been changed into log unit in the revised manuscript.**
